# Biocompatibility of cerium dioxide and silicon dioxide nanoparticles with endothelial cells

**DOI:** 10.3762/bjnano.5.190

**Published:** 2014-10-17

**Authors:** Claudia Strobel, Martin Förster, Ingrid Hilger

**Affiliations:** 1 Department of Experimental Radiology, Institute of Diagnostic and Interventional Radiology I, Jena University Hospital – Friedrich Schiller University Jena, Erlanger Allee 101, 07747 Jena, Germany; 2 Department of Internal Medicine I, Division of Pulmonary Medicine and Allergy/Immunology, Jena University Hospital – Friedrich Schiller University Jena, Erlanger Allee 101, 07747 Jena, Germany

**Keywords:** cerium dioxide, endothelial cells, nanoparticle, nanotoxicology, silicon dioxide

## Abstract

Cerium dioxide (CeO_2_) and silicon dioxide (SiO_2_) nanoparticles are of widespread use in modern life. This means that human beings are markedly exposed to them in their everyday life. Once passing biological barriers, these nanoparticles are expected to interact with endothelial cells, leading to systemic alterations with distinct influences on human health. In the present study we observed the metabolic impact of differently sized CeO_2_ (8 nm; 35 nm) and SiO_2_ nanoparticles (117 nm; 315 nm) on immortalized human microvascular (HMEC-1) and primary macrovascular endothelial cells (HUVEC), with particular focus on the CeO_2_ nanoparticles. The characterization of the CeO_2_ nanoparticles in cell culture media with varying serum content indicated a steric stabilization of nanoparticles due to interaction with proteins. After cellular uptake, the CeO_2_ nanoparticles were localized around the nucleus in a ring-shaped manner. The nanoparticles revealed concentration and time, but no size-dependent effects on the cellular adenosine triphosphate levels. HUVEC reacted more sensitively to CeO_2_ nanoparticle exposure than HMEC-1. This effect was also observed in relation to cytokine release after nanoparticle treatment. The CeO_2_ nanoparticles exhibited a specific impact on the release of diverse proteins. Namely, a slight trend towards pro-inflammatory effects, a slight pro-thrombotic impact, and an increase of reactive oxygen species after nanoparticle exposure were observed with increasing incubation time. For SiO_2_ nanoparticles, concentration- and time-dependent effects on the metabolic activity as well as pro-inflammatory reactions were detectable. In general, the effects of the investigated nanoparticles on endothelial cells were rather insignificant, since the alterations on the metabolic cell activity became visible at a nanoparticle concentration that is by far higher than those expected to occur in the in vivo situation (CeO_2_ nanoparticles: 100 µg/mL; SiO_2_ nanoparticles: 10 µg/mL).

## Introduction

Nowadays, a large variety of nanoparticles are being produced for different applications. These include the industrially and environmentally highly relevant cerium dioxide (CeO_2_) and silicon dioxide (SiO_2_) nanoparticles. CeO_2_, a rare-earth lanthanide element oxide, is mainly used in slurries for silicon wafer planarization [1–2], as automotive fuel additives to improve the efficiency of combustion [3–4], and as automobile catalytic converters [5]. SiO_2_ nanoparticles are employed in the fabrication of electric and thermal insulators [6], as drug-delivery systems in nanomedicine [7–8], as anticaking and thickener agents in food production [9–10], as well as in cosmetics, drugs and printer toners [11].

Human exposure to these nanoparticles arises not only from the consumption of products containing them, but also from their presence in the environment. Despite their widespread utilization, there is still uncertainty in the safety of these nanoparticles on human health, because appropriate experimental data are often contradictory. For example, it was shown that SiO_2_ nanoparticles can lead to pulmonary and cardiovascular alterations [12]. After inhalation in rats, they were shown to cause pulmonary inflammation, atrio-ventricular blockage, myocardial ischemic damage, increased blood viscosity [12] or lung fibrogenesis [13]. Moreover, SiO_2_ nanoparticles affect the protein expression of HaCaT cells [14]. In contrast, only low cytotoxicity to the human alveolar epithelial cell line A549, the human monocytic leukemia cell line THP-1 [15] or to the yeast *Saccharomyces cerevisiae* [6] was observed.

With respect to the CeO_2_ nanoparticles, several studies reported the presence of anti-oxidative [16–19], neuroprotective [20], cardioprotective [21], anti-inflammatory [22] and radioprotective properties [23]. Moreover, CeO_2_ nanoparticles fostered wound healing in mice due to reduction of oxidative damage [24]. However, in other studies an increase in oxidative stress after CeO_2_ nanoparticles exposure was shown [25–27].

Under certain circumstances nanoparticles can pass specific biological barriers (e.g., skin via wounds or lesions) and ultimately enter the blood vessel system. In consequence, interactions between endothelial cells and nanoparticles are possible with the consequence of cell death, inflammation and cardiovascular diseases. In this context, there is very little data available on the effects of these nanoparticles related to endothelial cells.

Therefore our aim was to clarify the impact of these different environmentally and industrially relevant nanoparticles on endothelial cells. We looked for size-dependent effects of CeO_2_ nanoparticles on endothelial cells. In particular we determined the relative cellular adenosine triphosphate (ATP) level to assess the cytotoxic potential of the nanoparticles, together with the pro-inflammatory response of exposed cells, and formation of reactive oxygen species (ROS). Furthermore, we also looked for effects of SiO_2_ nanoparticles on endothelial cells. Moreover, we considered if the nanoparticles’ effects on an immortalized cell line are comparable to a primary one.

## Results and Discussion

### CeO_2_ nanoparticle characterization

The smaller CeO_2_ nanoparticles (sample #A) exhibit a spherical shape with an average size of 8 nm as detected by transmission electron microscopy (TEM) (Table 1). The larger CeO_2_ nanoparticles (sample #B) were slightly elliptical and octahedral with an average circumscribed sphere diameter of 35 nm (Table 1). Both nanoparticle formulations had no surface coatings. The hydrodynamic diameters of both CeO_2_ nanoparticle samples were smaller in Millipore water than in cell culture medium (Table 1). This finding could be explained by the adsorption of the ions and proteins which are present in the culture medium. With increasing fetal bovine serum (FBS; protein) content in the cell culture medium, a smaller hydrodynamic diameter was observed (smaller nanoparticles, sample #A) shortly after preparation and also after 3 h of incubation time. This effect remains for steric stabilization in presence of serum proteins [28]. After a 3 h incubation of nanoparticles in culture medium (0.2%, 2% and 10% FBS), the diameters increased (for both nanoparticle samples), indicating some tendencies for nanoparticle agglomeration and aggregation with increasing time. The ζ-potential of the nanoparticles turned from positive values after suspension in Millipore water (sample #A: 23.2 mV; sample #B: 6 mV) to negative (−22.6 to −29.9 mV) when transferred to cell culture medium (0.2%, 2% and 10% FBS). This is also an indication of the adsorption of proteins from the cell culture media. Hereby, the ζ-potentials were neither positively nor negatively charged enough to prevent agglomeration by van der Waals forces [29]. Interestingly, the smaller nanoparticles (sample #A) exhibited a tendency towards an increasing negative charge with increasing serum content, while the larger nanoparticles (sample #B) revealed the opposite effect. This could be explained by differences in the extent or nature of nanoparticle–protein interactions in relation to nanoparticle size.

**Table 1:** Characterization of the CeO_2_ nanoparticles.

CeO_2_ nanoparticle	#A		#B	

Shape	spherical		elliptical and octahedral	
Diameter by TEM [nm]	8 ± 2		35 ± 10	
Diameter in H_2_O by DLS [nm]	74 ± 2 (PDI: 0.352)		163 ± 59 (PDI: 0.397)	

	shortly after preparation	3 h incubation	shortly after preparation^a^	3 h incubation^a^

Diameter in low serum cell culture medium (0.2% FBS) by DLS [nm]	449 ± 22 (PDI: 0.497)	606 ± 30 (PDI: 0.458)	261 ± 189	515 ± 54
Diameter in low serum cell culture medium (2% FBS) by DLS [nm]	427 ± 11 (PDI: 0.338)	598 ± 52 (PDI: 0.347)	188 ± 103	287 ± 87
Diameter in serum-rich cell culture medium (10% FBS) by DLS [nm]	251 ± 2 (PDI: 0.320)	304 ± 11 (PDI: 0.349)	223 ± 170	462 ± 221
ζ-potential in H_2_O [mV]	23.2 ± 0.9		6.0 ± 0.4	
ζ-potential in low serum cell culture medium [mV] (0.2% FBS)	−26.9 ± 0.4		−29.9 ± 0.4	
ζ-potential in low serum cell culture medium (2% FBS) [mV]	−27.9 ± 0.3		−28.9 ± 0.2	
ζ-potential in serum-rich cell culture medium (10% FBS) [mV]	−29.6 ± 0.1		−22.6 ± 0.5	
TEM pictures	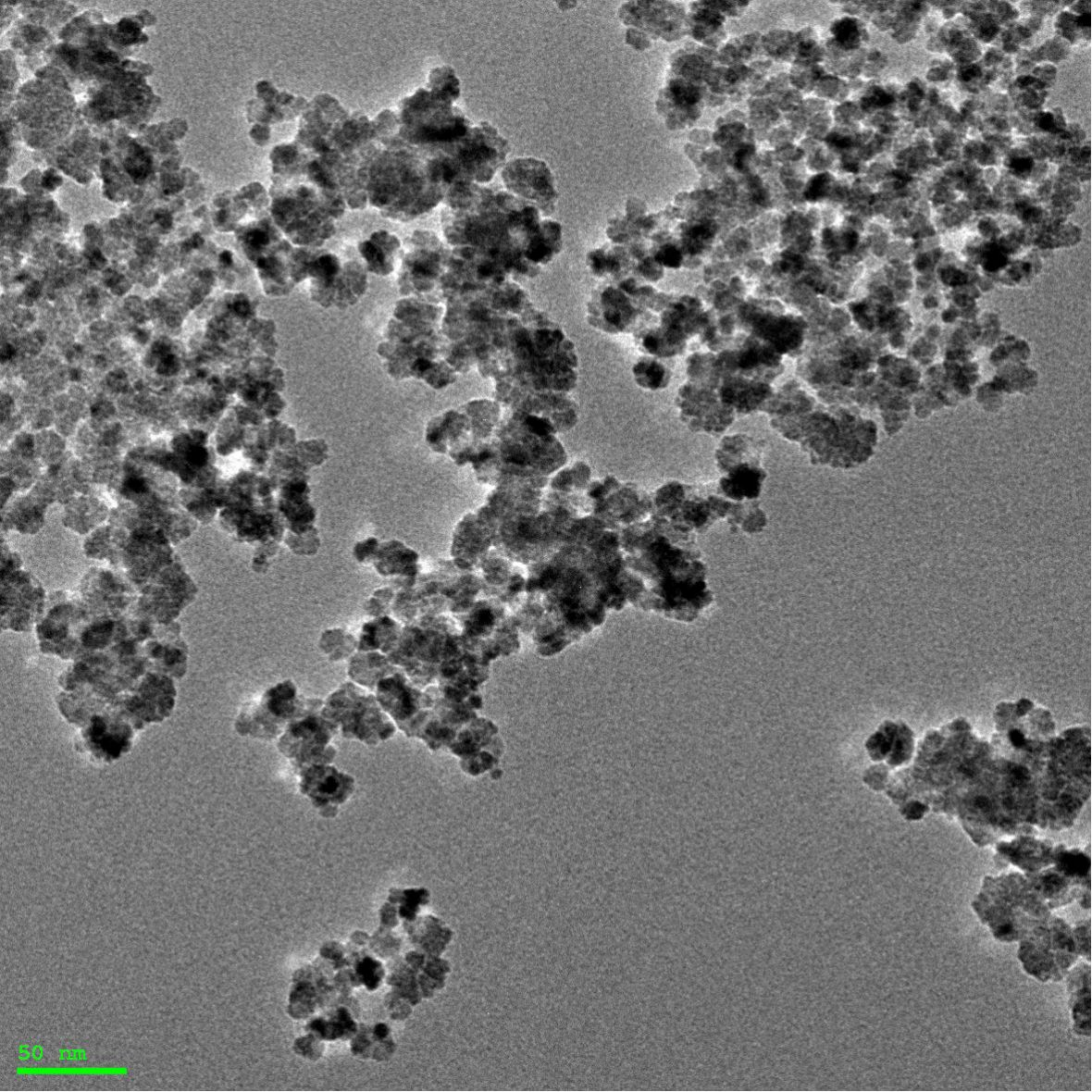		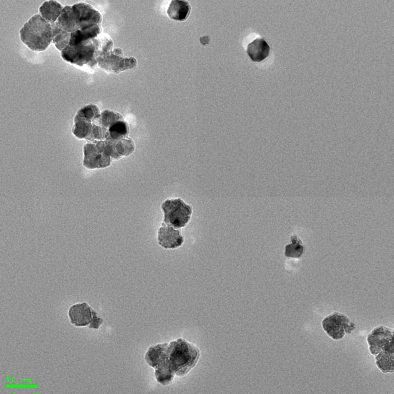	

^a^The polydispersity index (PDI) was over 0.5, therefore the diameters were determined by distribution analysis.

### Characterization of the endothelial phenotype of target cells

**Figure 1:**
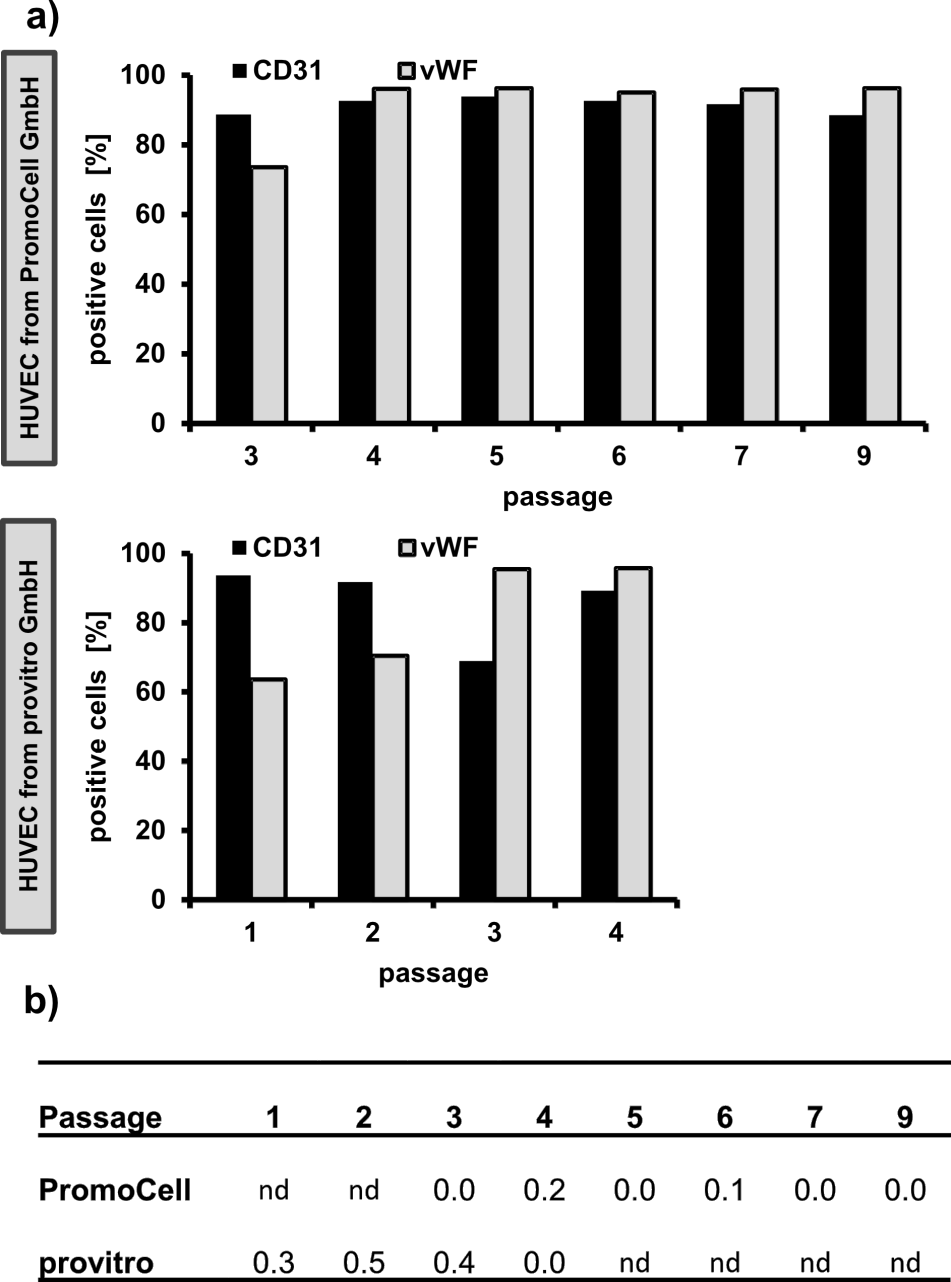
The HUVEC populations were pure and retained the endothelial phenotype during the experiment. a) Proportion of CD31^+^ and vWF^+^ cells [%]. The endothelial phenotype was determined via the detection of von Willebrand factor (vWF) and CD31 (platelet endothelial cell adhesion molecule 1 (PECAM-1)). b) Proportion of CD90^+^ cells [%]. For purity examinations, CD90 as fibroblast cell type specific marker was determined. The investigations were performed on up to 9 passages and on HUVEC from two different suppliers (PromoCell GmbH and provitro GmbH). nd: not determined.

In addition to the study of the impact of nanoparticles on human immortalized endothelial cells (HMEC-1), primary endothelial cells (human umbilical vein endothelial cell HUVEC, PromoCell GmbH, Germany) were also used. The aim was to assess if there are differences in the sensitivity of these cell types which are detectable after exposure to the nanoparticles. Since HUVEC were isolated from the vein of an umbilical cord, the presence of fibroblasts cannot be excluded [30]. Moreover, primary cells are known to change their phenotype with increasing cultivation time [31]. In this context, the assessment of the endothelial phenotype with respect to cultivation time was of interest. The investigated HUVEC populations presented an endothelial phenotype up to the highest investigated passage number as can be seen in Figure 1. All passages showed mainly CD31^+^ (platelet endothelial cell adhesion molecule 1 (PECAM-1)) and von Willebrand factor (vWF) positive cells (endothelial cells, Figure 1a) and nearly no CD90^+^ cells (fibroblasts, Figure 1b). vWF and CD31 are known to be endothelial [32] and CD90 is a fibroblast cell type specific marker [33]. The experiment was successfully validated using HUVEC from another supplier (provitro GmbH, Germany; Figure 1). Reactivity of CD90 antibody against CD90^+^ human fibroblasts (BJ-cells) was corroborated in a previous experiment (≥99% CD90^+^ were detected, data not shown). It can be concluded that the HUVEC culture was pure with no alterations of the endothelial phenotype during the experimental setup. This means that the obtained results of the present study truly reflect the response of endothelial cells after nanoparticle exposure.

### Intracellular localization of CeO_2_ nanoparticles

**Figure 2:**
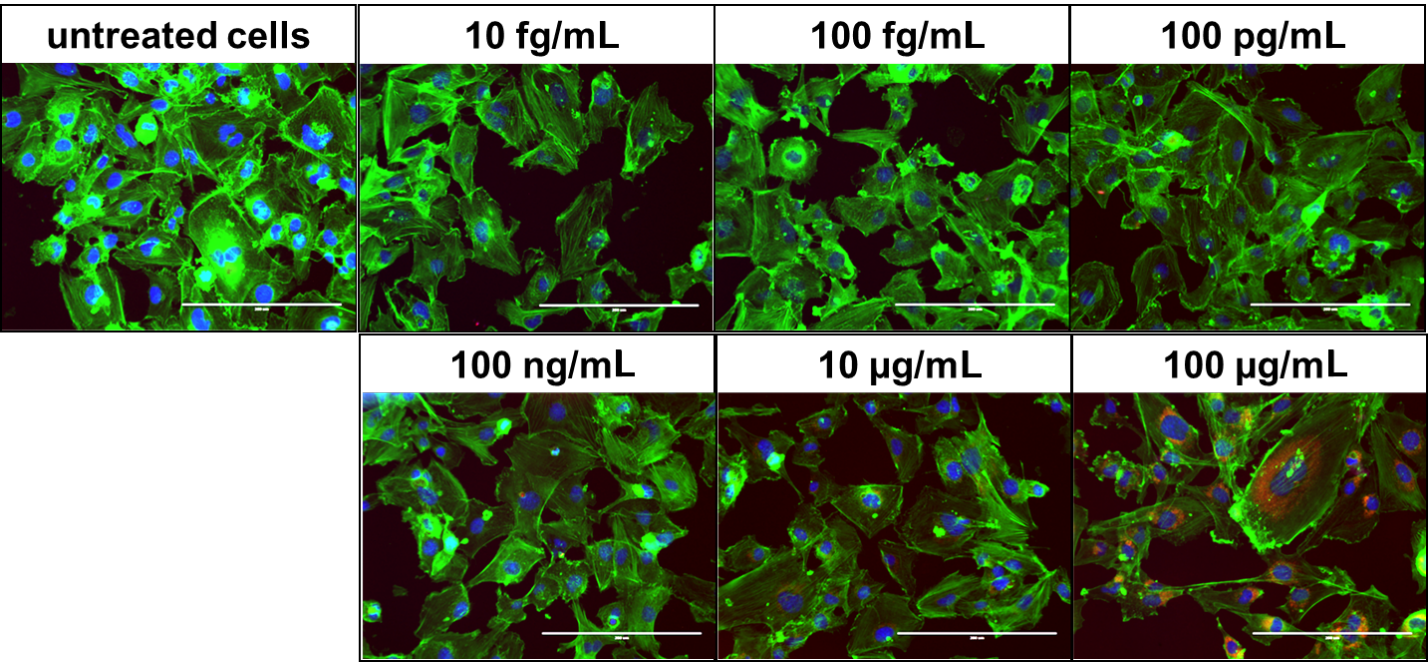
CeO_2_ nanoparticles were localized peri-nuclearly within endothelial cells. HMEC-1 were exposed to different concentrations of CeO_2_ nanoparticles (sample #B, 35 nm) for 48 h. blue: nucleus (Hoechst); green: actin (Alexa Fluor^®^ 546 Phalloidin); red: CeO_2_ nanoparticles (*N*-(2,5-bis(dimethylethyl)phenyl)-*N*’-(3-(triethoxysilyl)propyl)perylene-3,4,9,10-tetracarboxylic acid diimide label); magnification: 20×.

It was found that the investigated CeO_2_ nanoparticles were taken up by endothelial cells (HMEC-1) and that they were localized in a ring-shape around the nucleus in an aggregated manner (Figure 2; Supporting Information File 1, Figure S1). An investigation with another cell type is in agreement with this observation (human lung epithelial cells (BEAS-2B); after exposure to 30 nm diameter CeO_2_ nanoparticles) [27]. A peri-nuclear localization of nanoparticles is also known for other metal oxide nanoparticles, such as TiO_2_ nanoparticles [34] or iron oxide nanoparticles [35]. This indicates that the peri-nuclear accumulation is not dependent on the nanoparticle chemistry.

Although the concentration-dependent nanoparticle exposure revealed no obvious differences in the cell morphology (48 h; Figure 2), direct interactions of the internalized nanoparticles with specific molecules during intracellular processing and degradation are quite possible, particularly because of the peri-nuclear localization in the cytoplasm, which might correspond to the endoplasmic reticulum. These relationships could explain, at least partially, the encountered effects on cell metabolism (cellular ATP levels, pro-inflammatory reactions etc.) described below.

### Impact of CeO_2_ nanoparticle exposure on the metabolic activity of endothelial cells

#### Impact of CeO_2_ nanoparticles on cellular ATP level

**Figure 3:**
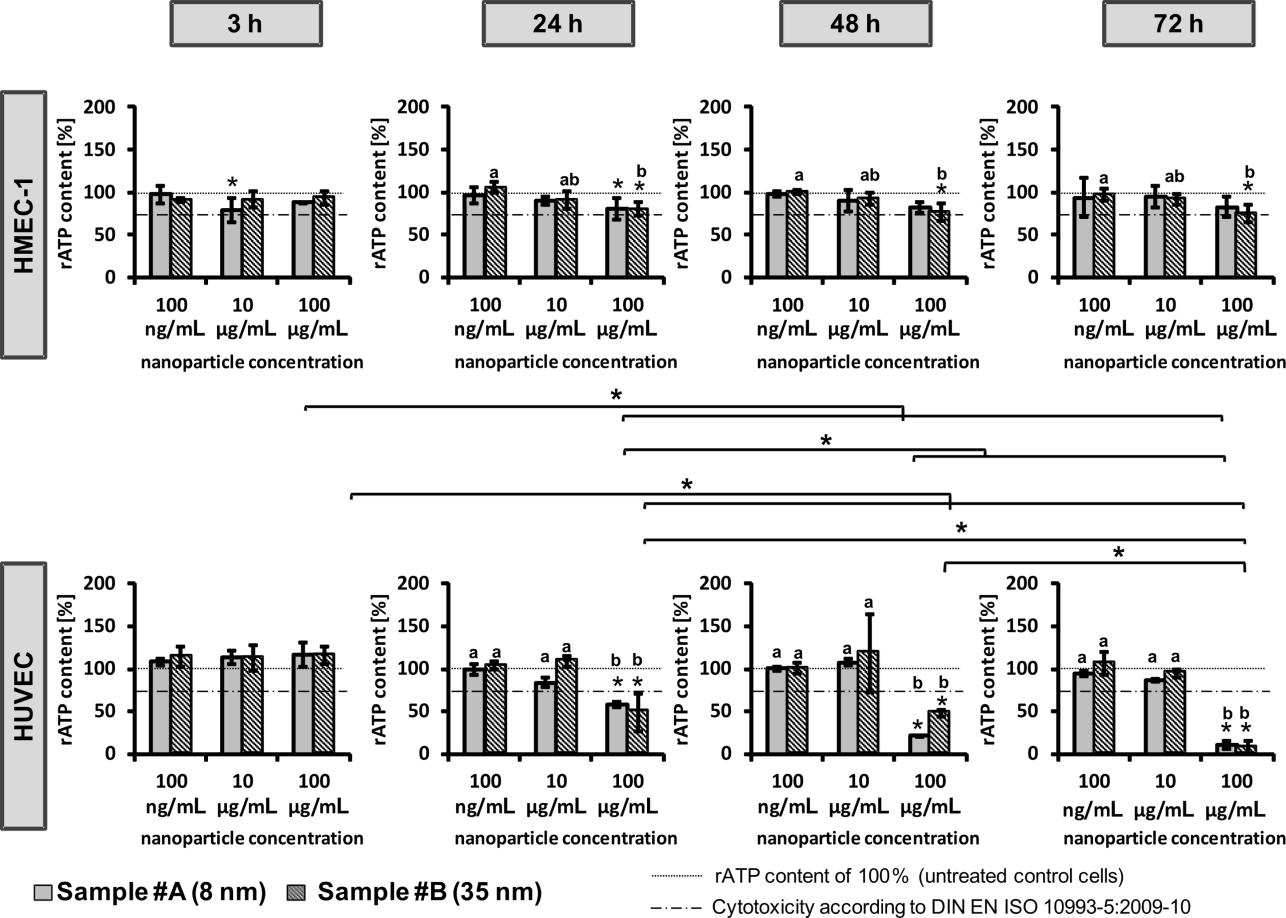
CeO_2_ nanoparticles revealed concentration- and time-dependent effects on the cellular adenosine triphosphate (ATP) level. Immortalized human microvascular endothelial cells (HMEC-1) and primary human macrovascular endothelial cells (HUVEC) were exposed to CeO_2_ nanoparticles of different sizes (sample #A (8 nm), sample #B (35 nm)), concentrations, and incubation times (3 h, 24 h, 48 h, 72 h). rATP content: relative ATP content; *n* = 3 independent experiments; *single asterisks over the bar indicate significant differences (P ≤ 0.05) between the relative ATP content of cells after treatment with the corresponding nanoparticle concentration and the relative ATP content of 100% (untreated control cells); a, b, c indicate significant differences (P ≤ 0.05) of one nanoparticle formulation among different concentrations; *asterisks, which are together with a parenthesis, indicate significant differences (P ≤ 0.05) between time points for a specific concentration of one nanoparticle formulation.

The cellular ATP content was determined as a measure of the metabolic activity of endothelial cells after nanoparticle treatment and the cytotoxic potential of the nanoparticles. In general, the small- (sample #A) and large-sized (sample #B) nanoparticles induced comparable effects (Figure 3). However, a distinct concentration dependence was observed. In particular, a high nanoparticle concentration of 100 µg/mL led to a decrease of the cellular ATP levels with increasing incubation times, which was most prominent for the primary endothelial cells (HUVEC; Figure 3).

It should be taken into account that the concentration of 100 µg/mL is physiologically unrealistic and cannot be reached in vivo. It is conceivable that in addition to the direct nanoparticle impact on cells, that unspecific effects due to an overloading of the cells with nanomaterial could occur [36]. This would lead to depletion of nutrients and oxygen. Nevertheless, according to Thomassen et al. [37] we expect that this influence is rather low. ATP values lower than the threshold for cytotoxicity (according to DIN EN ISO 10993-5:2009-10, distinct cytotoxic effects) were observed only for HUVEC. In comparison, CeO_2_ nanoparticles were also found to be cytotoxic in other cell types, such as human bronchial epithelial cells [25,27] or human lung cancer cells [26]. The extent of the adverse effects of CeO_2_ nanoparticles on cells seems to be cell type-dependent. This applies also for subsets of endothelial cells which have been derived from different tissue types. Interestingly, the gene expression profiles of microvascular and macrovascular endothelial cells are different between each other; the expression patterns are also determined by the respective tissue from which they have been derived [38–39]. Therefore, it is conceivable that the observed differences in sensitivity of the two endothelial cell types in our study (HMEC-1: immortalized, microvascular; HUVEC: primary, macrovascular) to CeO_2_ nanoparticle exposure is a result of different gene expression patterns.

Furthermore, the difference in the stability of actin filaments between microvascular (HMEC-1) and macrovascular endothelial cells (HUVEC) [39–40] could explain the different behavior mentioned above. It is conceivable that stable actin filaments (HMEC-1) avoid disturbance of the cellular machinery, which might be induced by CeO_2_ nanoparticles. Owing to comparable doubling times of the corresponding endothelial cells (HMEC-1: approximately 33.6 h; HUVEC: approximately 36 h), it can be excluded that cell division caused the observed differences in the sensitivity of the cells on nanoparticle treatment.

The use of primary and immortalized endothelial cell lines in cytotoxicity examinations has a series of advantages and disadvantages. In particular, the phenotype of HUVEC should resemble the in vivo situation to a higher extent than immortalized ones, but require specific culture media conditions and life span in culture is limited. Immortalized cell lines are advantageous for cytotoxicity screening, since they are easy to handle. According to the results described above, it is important to use not only immortalized but also primary endothelial cells for studying the cellular effect of nanoparticles in order to get a comprehensive picture.

#### Pro-inflammatory and pro-thrombotic impact of CeO_2_ nanoparticles and intracellular ROS generation after CeO_2_ nanoparticle exposure

**Figure 4:**
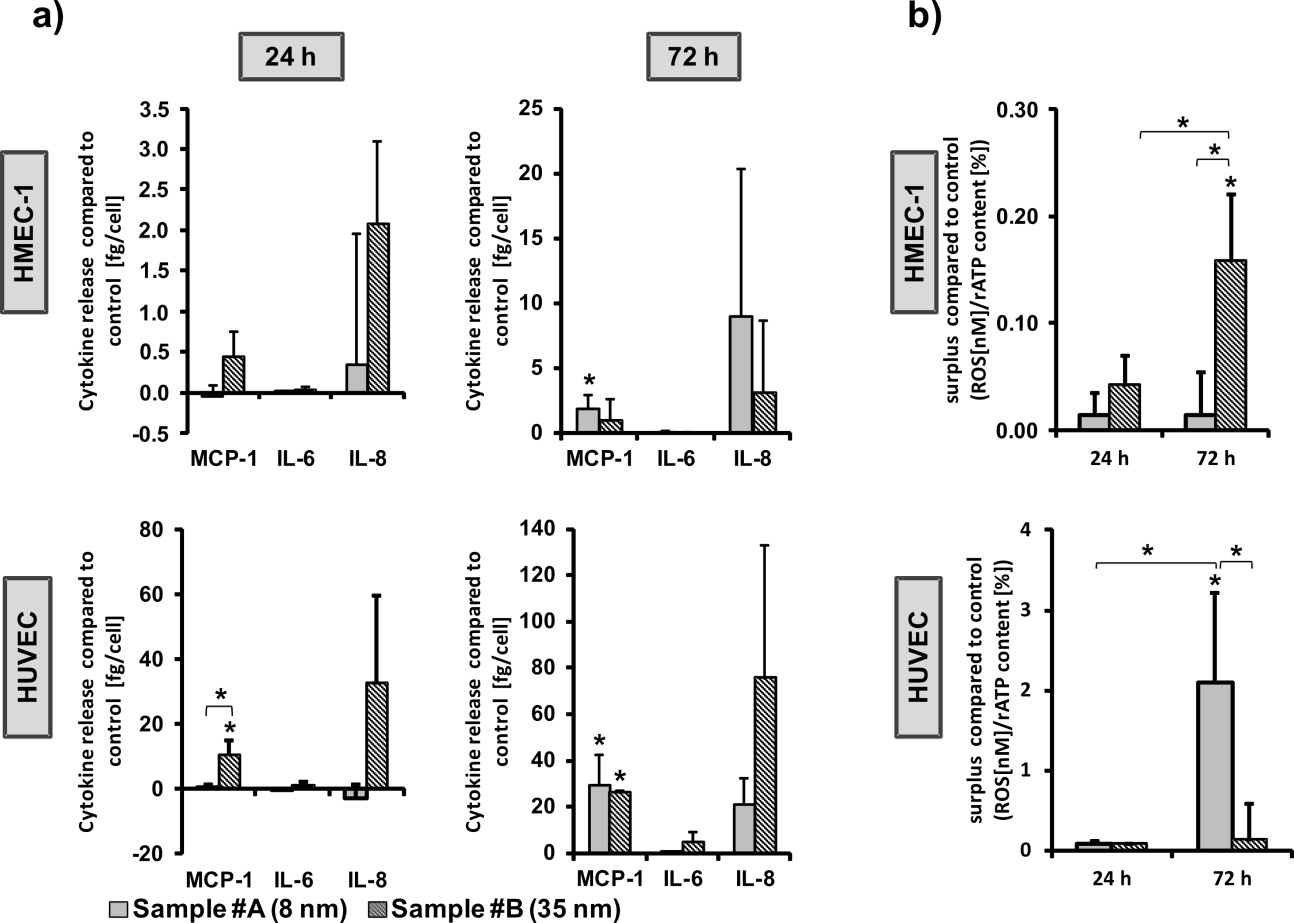
Pro-inflammatory impact and ROS generation of CeO_2_ nanoparticle exposure on endothelial cells. a) MCP-1, IL-6 and IL-8 release after nanoparticle treatment (100 µg/mL) at different nanoparticle exposure times (24 h; 72 h) are shown for HMEC-1 and HUVEC; data depicted as the difference between values after treatment and untreated controls (surplus compared to control). Values of the positive controls for the pro-inflammatory effects and the absolute values of the untreated control cells are found in the Supporting Information File 1 (Figure S2 and Figure S3, respectively). b) Intracellular ROS production after CeO_2_ nanoparticle treatment, data depicted as the difference between values of treated and untreated control cells (surplus compared to control). ROS = reactive oxygen species; *n* = 3 independent experiments; *single asterisks over the bar indicate significant differences (P ≤ 0.05) between the appropriate treatment and untreated control cells; *asterisks, which are together with a parenthesis, indicate significant differences (P ≤ 0.05) between different nanoparticle formulations or between different time points.

In order to identify the pro-inflammatory impact of CeO_2_ nanoparticles, the release of three different cytokines (monocyte chemoattractant protein-1 (MCP-1), interleukin 6 (IL-6), and IL-8) as pro-inflammatory markers after CeO_2_ nanoparticle treatment were determined. After a 24 h exposure time, cells treated with smaller nanoparticles (sample #A) tended to induce a lower cytokine release compared to cells treated with larger nanoparticles (sample #B; Figure 4a). After 72 h of incubation, both investigated CeO_2_ nanoparticle formulations caused an increase of cytokine release (Figure 4a), particularly of MCP-1 and IL-8, which act as chemo-attractants for monocytes or neutrophils and T lymphocytes during the development of chronic inflammation [41–42]. The IL-6 release after nanoparticle treatment was only marginal compared to untreated controls.

Since it is known that reactive oxygen species (ROS) can activate distinct signaling pathways leading to inflammatory cytokine up-regulation [43], the differences between the impact of small- (sample #A) and large-sized (sample #B) CeO_2_ nanoparticles on the cytokine release could theoretically be associated with the production of ROS. Additionally, the observed ROS generation correlates with the cytokine release of HMEC-1 after 24 h of incubation. Since this was not the case after 72 h, a short-term effect of ROS on the pro-inflammatory response machinery may be postulated. In HUVEC, no correlation between the ROS generation and the cytokine release was detectable. Hereto, other mechanisms seem to be responsible for these processes.

Interestingly, the quantification of intracellular CeO_2_ nanoparticles (sample #A and #B) in HMEC-1, as was investigated by A. A. Torrano et al. [44] is in agreement with the observed ROS production pattern. Therefore, the sample-mediated differences in ROS production could be attributed to different amounts of internalized nanoparticles, depending on the nanoparticle size.

Obviously, the aforementioned effects are cell type-dependent. In particular in HMEC-1, large-sized CeO_2_ nanoparticles (sample #B) revealed a larger impact on the ROS generation than small-sized ones (sample #A), whereas in HUVEC the opposite was observed. Data in the literature are conflicting regarding the ROS generation of CeO_2_ nanoparticles. Several studies reported either anti-oxidative properties or an increase of oxidative stress. In particular 8 nm-sized CeO_2_ nanoparticles suppressed ROS production [45], while 30 nm-sized nanoparticles induced oxidative stress in human bronchial epithelial cells (Beas-2B) [25]. Therefore, general predictions are not possible at present. Nevertheless, the different behavior could explained, at least in parts, by the exposure of different intracellular nanoparticle amounts per cell as a result of cell type specific variations in cellular uptake and exocytosis rates.

**Figure 5:**
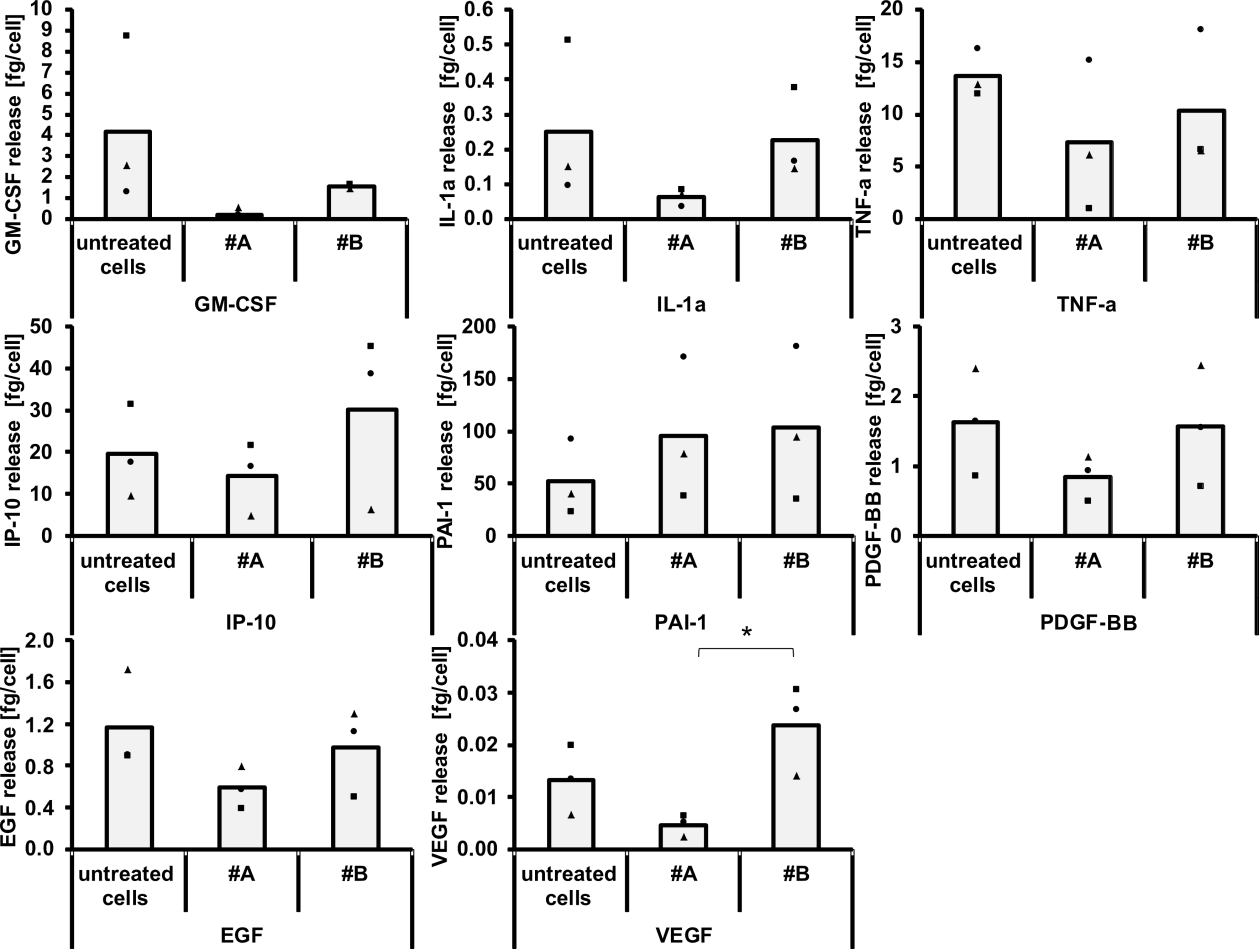
The impact of CeO_2_ nanoparticles on the release of GM-CSF, IL-1α, TNF-α, IP-10, PAI-1, PDGF-BB, EGF and VEGF. HUVEC were treated with CeO_2_ nanoparticles (sample #A or sample #B, see text; 100 µg/mL) for 24 h. *n* = 3 independent experiments; *asterisks indicate significant differences (P ≤ 0.05).

We also investigated the release of granulocyte macrophage colony-stimulating factor (GM-CSF), interleukin-1 α (IL-1α), tumor necrosis factor α (TNF-α), interferon gamma-induced protein 10 (IP-10), plasminogen activator inhibitor-1 (PAI-1), platelet-derived growth factor (PDGF-BB), epidermal growth factor (EGF) and vascular endothelial growth factor (VEGF) of HUVEC exposed to CeO_2_ nanoparticles for 24 h (Figure 5). In general, the release of these proteins was lowest after treatment with the small-sized CeO_2_ nanoparticles (sample #A) compared to their large-sized counterparts (sample #B). This could be caused, at least in part, by protein adsorption on the nanoparticle surface, which would be higher for the small-sized nanoparticles as a result of a higher surface–volume relationship [46]. This could ultimately lead to a distinct impact on cell metabolism and cell–cell interactions. Both nanoparticle samples showed the tendency of an increase of PAI-1. The effects are the same for a slight pro-thrombotic impact of CeO_2_ nanoparticles, since increased PAI-1 plasma levels are related to a risk of atherothrombosis development [47–48]. Importantly, the large-sized nanoparticles (sample #B) increased the release of IP-10. This protein is related to the recruitment of activated T cells [49], which is a contribution to inflammative processes [50]. Moreover, large-sized CeO_2_ nanoparticles (sample #B) led to an increase of VEGF release, which is widely known to act as a potent angiogenesis stimulus [51]. This would mean that large-sized CeO_2_ nanoparticles (sample #B) are able to promote angiogenesis, at least in parts. A tendency towards decreased levels of the pro-inflammatory markers MCP-1, IL-8 (Figure 4a), GM-CSF, IL-1α, TNF- α, IP-10, as well as of the growth factors EGF, VEGF and PDGF-BB (Figure 5) were seen in relation to the small-sized CeO_2_ nanoparticles (sample #A, 24 h of incubation, HUVEC). The findings demonstrate the complexity of reactions in terms of protein biosynthesis and protein release – even alterations of the cellular vesicular transport are conceivable. It cannot be excluded that cell material of dead cells could partly affect the determined cytokine release.

### Impact of SiO_2_ nanoparticles on endothelial cells

**Figure 6:**
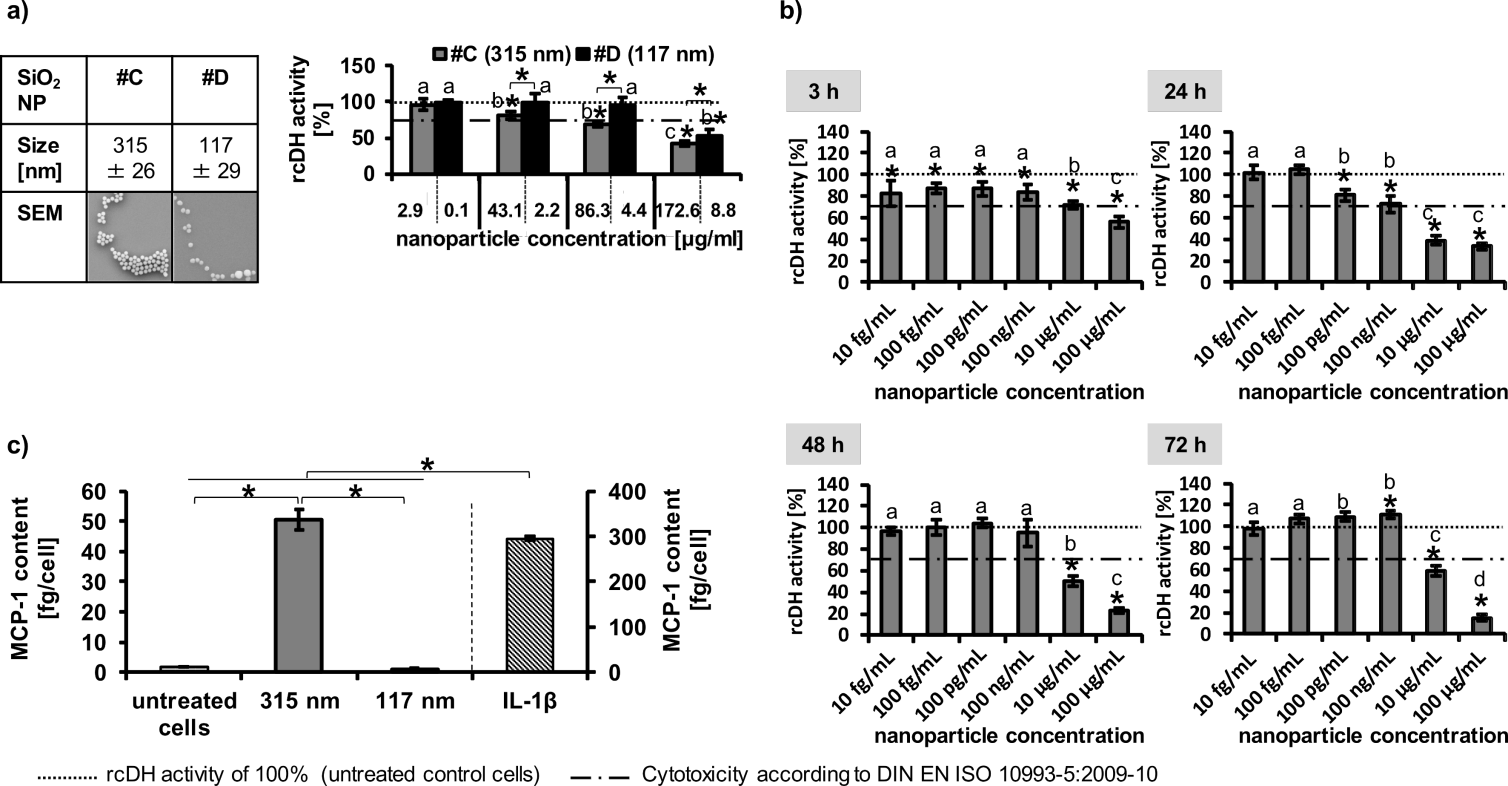
Metabolic impact of SiO_2_ nanoparticles on endothelial cells. a) Impact of two different sized SiO_2_ nanoparticles on HUVEC after 24 h (*n* = 6). The different concentrations between the two different sized nanoparticles correspond to equal nanoparticle number per seeded cells (1,000 nanoparticles per seeded cell: nanoparticle sample #C: 2.9 µg/mL, sample #D: 0.1 µg/mL; 15,000 nanoparticles per seeded cell: sample #C: 43.1, sample #D: 2.2; 30,000 nanoparticles per seeded cell: sample #C: 86.3 µg/mL, sample #D: 4.4 µg/mL; 60,000 nanoparticles per seeded cell: sample #C: 172.6 µg/mL, sample #D: 8.8 µg/mL). b) Impact of 315 nm SiO_2_ nanoparticles (sample #C) on HMEC-1 (*n* = 6). c) The pro-inflammatory impact of SiO_2_ nanoparticles (30,000 nanoparticles per cell [sample #C: 86.3 µg/mL; sample #D: 4.4 µg/mL]; 24 h incubation; HUVEC; *n* = 3) was determined via MCP-1 release. IL-1β served as positive control to test the ability of cells for cytokine release after treatment with an appropriate stimulus (*c* = 2000 pg/mL). *Single asterisks over the bar indicate significant differences (P ≤ 0.05) between the relative cellular dehydrogenase (rcDH) activity of cells after treatment with the corresponding nanoparticle concentration and the rcDH activity of 100% (untreated control cells); a, b, c indicate significant differences (P ≤ 0.05) of one nanoparticle formulation among different concentrations; *asterisks, which are together with a parenthesis, indicate significant differences (P ≤ 0.05) between different nanoparticle formulations.

We also investigated the impact of SiO_2_ nanoparticles on endothelial cells (Figure 6). A concentration- (Figure 6a,b), size- (based on the same nanoparticle number per seeded cell; Figure 6a), and exposure time-dependence (Figure 6b) was observed in relation to their impact on the cellular dehydrogenase (rcDH) activity. The different concentrations between the two different-sized nanoparticles in Figure 6a correspond to equal nanoparticle numbers per seeded cells (e.g., 1,000 nanoparticles per seeded cell: nanoparticle sample #C: 2.9 µg/mL, sample #D: 0.1 µg/mL; 15,000 nanoparticles per seeded cell: sample #C: 43.1, sample #D: 2.2; 30,000 nanoparticles per seeded cell: sample #C: 86.3 µg/mL, sample #D: 4.4 µg/mL; 60,000 nanoparticles per seeded cell: sample #C: 172.6 µg/mL, sample #D: 8.8 µg/mL). Even if the diameters of the SiO_2_ and CeO_2_ nanoparticles are not comparable, it can be seen that in contrast to the CeO_2_ nanoparticles, HMEC-1 cells showed a higher sensitivity to SiO_2_ than the HUVEC. However, the comparison between HMEC-1 and HUVEC was only studied after 24 h and not for longer time periods (Figure 6).

The assessment of the pro-inflammatory impact of SiO_2_ nanoparticles (30,000 nanoparticle per cell; 24 h incubation; HUVEC) revealed a size dependency (Figure 6c). For this investigation we normalized the nanoparticle amount per seeded cell independent of the nanoparticle size as we did also for the impact on the cellular dehydrogenase activity when comparing the different sized nanoparticles. If we calculate the corresponding concentrations in µg/mL, the cells treated with 315 nm-sized nanoparticles (sample #C) were exposed to a higher nanoparticle concentration (86.3 µg/mL) than cells incubated with 117 nm-sized particles (sample #D; 4.4 µg/mL). Therefore, the size-dependent effect on the MCP-1 release (Figure 6c) and dehydrogenase activity (Figure 6a) could be a result of the different material concentrations.

Taken together, our in vitro investigations revealed distinct effects of CeO_2_ and SiO_2_ nanoparticles on human primary macrovascular as well as on immortalized microvascular endothelial cells.

However, considering the concentrations which would be achieved after exposure of endothelial cells in vivo, the impact of CeO_2_ and SiO_2_ nanoparticles should be rather low as adverse effects were only observed at high concentrations, which overestimate realistic concentrations in the in vivo situation. In particular, if we consider an exposure of human beings to CeO_2_ nanoparticles in areas of high traffic with a concentration of 1 ng CeO_2_/m^3^ air [52] and suppose that all inhaled nanoparticles per day translocate to the blood stream, we would find a concentration of 0.0003 fg CeO_2_/cm^2^ endothelial surface in vivo instead of 2.9 μg/cm^2^ endothelium as applied in vitro. The latter mentioned concentration corresponds to a non-toxic value of 10 µg/mL as it was applied in vitro. Thus, the expected in vivo effects of the investigated nanoparticles should be low, but this finding must be verified by in vivo studies.

## Conclusion

Our in vitro study contributes to a better understanding of the impact of CeO_2_ and SiO_2_ nanoparticles on isolated endothelial cells, particularly due to inclusion of microvascular and primary macrovascular endothelial cells. In particular, we observed distinct effects depending on the cell type (immortalized microvascular vs primary macrovascular endothelial cells), nanoparticle formulation (CeO_2_, SiO_2_ nanoparticles), concentration, exposure time and nanoparticle size. In this context, differently sized CeO_2_ nanoparticles revealed different effects on the release of pro-inflammatory, pro-thrombotic markers and growth factors. Primary macrovascular endothelial cells reacted more sensitively to CeO_2_ nanoparticles than immortalized microvascular endothelial cells. The intracellular ROS generation was not only dependent on nanoparticle size, but also on cell type due to potential differences in nanoparticle uptake and retention rates (CeO_2_ nanoparticles). With consideration of the expected nanoparticle concentrations in endothelial cells in vivo, the impact of CeO_2_ and SiO_2_ nanoparticles can be considered as rather low.

## Experimental

### Nanoparticles used in this study

The synthesis of CeO_2_ nanoparticles based on the principle of Chen and Chang [53–54] and is described by Herrmann et al. [55]. SiO_2_ nanoparticle samples were synthesized as described previously [56].

The SiO_2_ nanoparticles were stored in water and the CeO_2_ nanoparticles were stored in ethanol as a solvent. Before starting the experiments, the ethanol was replaced by Millipore water by four centrifugation/redispersion (1.0 mL water) steps. To prepare nanoparticle working suspensions, the stock suspensions were vortexed and placed in an ultrasound bath (Bandelin Sonorex RK 52 H, Bandelin electronic GmbH & Co. KG, Germany; HF-power: 60 W (effective)) for 10 min.

### Nanoparticle characterization

Transmission electron microscopy (TEM) measurements were carried out to determine the size and shape of the nanoparticle samples.

The measurements of the hydrodynamic diameters and the ζ-potentials of the CeO_2_ nanoparticles were conducted using a zetasizer instrument (Nano ZS Malvern Instruments, UK). For these measurements, the concentration of the CeO_2_ nanoparticle suspensions was 50 µg/mL either in Millipore water or cell culture media (Gibco^®^ MCDB 131 medium (Life Technologies GmbH, Germany; supplemented with fetal bovine serum (FBS, 10% or 0.2% (v/v), Life Technologies GmbH, Germany), GlutaMAX^TM^ I 100X (1% (v/v), Life Technologies GmbH, Germany), hydrocortisone (1 µg/mL; Sigma-Aldrich Chemie GmbH, Germany)); or endothelial cell growth medium (Ready-to-use, PromoCell GmbH, Germany; supplemented with SupplementMix, PromoCell GmbH, Germany; FBS 2% (v/v))).

### Cell culture experiments

The experiments were performed with immortalized human microvascular endothelial cells (HMEC-1; Centers for Disease Control and Prevention, USA) and with primary human umbilical vein endothelial cells (HUVEC; PromoCell GmbH, Germany). Cultivation of HMEC-1 was performed using Gibco^®^ MCDB 131 medium (Life Technologies GmbH, Germany) supplemented with fetal bovine serum (FBS, 10% (v/v), Life Technologies GmbH, Germany), GlutaMAX^TM^ I 100X (1% (v/v), Life Technologies GmbH, Germany), hydrocortisone (1 µg/mL; Sigma-Aldrich Chemie GmbH, Germany) and epidermal growth factor (10 ng/mL; Life Technologies GmbH, Germany). HUVEC were cultivated in endothelial cell growth medium (Ready-to-use, PromoCell GmbH, Germany) supplemented with SupplementMix (PromoCell GmbH, Germany). Both cell lines were cultured at 37 ºC in a 5% CO_2_ humidified environment and the growth medium was exchanged every 2–3 days. Once the cells reached 70–85% confluency they were subcultivated. To detach the cells, GIBCO^®^ trypsin (Life Technologies GmbH, Germany) was used. The cells routinely tested negative for mycoplasma via PCR.

#### Characterization of HUVEC population via flow cytometry analysis

HUVEC are primary endothelial cells, which were isolated from the vein of an umbilical cord. To check the endothelial phenotype, flow cytometry analysis was conducted (FACS Calibur; Becton Dickinson GmbH, Germany; 488 nm and 635 nm lasers; filters: FI1 530/30; FI2 585/42; FI3 670 LP; FI4 661/16). Additionally, HUVEC from another supplier (provitro GmbH, Germany) were analyzed. vWF and CD31 were determined as endothelial and CD90 as fibroblast cell type specific markers, respectively. After staining both CD31 (monoclonal anti-human CD31 antibodies conjugated to fluorescein isothiocyanate (FITC), Miltenyi Biotec GmbH, Germany) and CD90 (monoclonal anti-human CD90 antibodies conjugated to R-phycoerythrin (PE) Miltenyi Biotec GmbH, Germany), the cells were washed with buffer (1% BSA [Albumin Fraktion V, Carl Roth GmbH & CO. KG, Germany] in Hank’s BSS [PAA Laboratories GmbH, Austria]). Then the cells were fixed with 2% (v/v) formaldehyde (Carl Roth GmbH & CO. KG, Germany) in Hank’s BSS for 15 min at room temperature. The cells were then washed with Hank’s BSS. As a permeabilization reagent, 0.1% (v/v) Saponin (Sigma-Aldrich Chemie GmbH, Germany) in Hank’s BSS was used. Intracellular staining of vWF with allophycocyanin (APC) conjugated mouse monoclonal anti-human vWF-A2 antibodies (R&D Systems, Inc., USA) followed. Unstained cells, cells stained with mouse IgG1 isotype control antibodies conjugated to FITC (Miltenyi Biotec GmbH, Germany), mouse IgG1 isotype control antibodies conjugated to PE (Miltenyi Biotec GmbH, Germany) or mouse IgG2B isotype control antibodies conjugated to APC (R&D Systems, Inc., USA) served as specificity controls. Human fibroblasts (BJ cells, American Type Culture Collection (ATCC), USA) were used as positive cells for CD90 (fibroblast phenotype). 10,000 cells were measured for each sample and analysis was performed using CellQuest Pro^TM^ software (Becton Dickinson GmbH, Germany).

#### Cellular uptake and intracellular localization of CeO_2_ nanoparticles

To semi-qualitatively assess the uptake and intracellular localization of the CeO_2_ nanoparticles, fluorescent microscopy (Evos fl; PEQLAB Biotechnologie GmbH, Germany) was used. HMEC-1 were exposed to different concentrations (10 fg/mL to 100 µg/mL) of CeO_2_ nanoparticles (35 nm; sample #B, labeled with the fluorescence marker *N*-(2,5-bis(dimethylethyl)phenyl)-*N*’-(3-(triethoxysilyl)propyl)perylene-3,4,9,10-tetracarboxylic acid diimide (MPD)) for 48 h. After a washing step with Hank’s BSS (PAA Laboratories GmbH, Austria), fixation with 3.7% (v/v) formaldehyde (Carl Roth GmbH & CO. KG, Germany) in Hank’s BSS for 10 min at 4 ºC was carried out. After washing with Hank’s BSS, the cells were permeabilized with 0.1% Triton X-100 (Sigma-Aldrich Chemie GmbH, Germany) in Hank’s BSS for 3 min. Once again the cells were washed with Hank’s BSS. The cellular F-actin was stained with Alexa-Fluor^®^-546 Phalloidin (5 units/ml; 20 min at room temperature; Life Technologies GmbH, Germany), and the cell nuclei with Hoechst 33258 (0.2 µg/mL; AppliChem GmbH, Germany). The cells were embedded in Permafluor^®^ (Thermo Fisher, USA) and analyzed via fluorescence microscopy (Evos fl; PEQLAB Biotechnologie GmbH, Germany; magnification: 20×).

#### Determination of relative cellular ATP level to assess the metabolic activity of endothelial cells after CeO_2_ nanoparticle treatment

HMEC-1 and HUVEC cultured in white 96-well culture plates were treated with different concentrations of CeO_2_ nanoparticles (100 ng/mL, 10 µg/mL, 100 µg/mL) for defined incubation times (3, 24, 48 and 72 h). Afterwards, the cells were washed with Hank’s BSS (PAA Laboratories GmbH, Austria) and the CellTiter-Glo^®^ Luminescent Cell Viability Assay (Promega GmbH, Germany) was carried out according to the manufacturer’s instructions. On the basis of the measured luminescence (LUMIStar Galaxy OPTIMA microplate reader, BMG LABTECH GmbH, Germany), the relative ATP content was calculated and normalized to corresponding untreated control cells. The threshold for cytotoxicity according to DIN EN ISO 10993-5:2009-10 was used as orientation to evaluate the results.

#### Assessment of the impact of the nanoparticles on the release of different proteins

To assess the pro-inflammatory impact of the CeO_2_ nanoparticles, HMEC-1 and HUVEC were treated with a concentration of 100 µg/mL of CeO_2_ nanoparticles for either 24 h or 72 h. Cells treated with interleukin 1β served as a positive control to test the ability of cells for cytokine release after treatment with an appropriate stimulus (IL-1β; *c* = 2000 pg/mL; data shown in Supporting Information File 1, Figure S2; Sigma-Aldrich Chemie GmbH, Germany). For HMEC-1, serum-reduced culture medium was used (0.2% FBS), since the serum itself could contain cytokines. After the corresponding incubation time, the cell culture supernatants were collected and stored at −80 °C until the human enzyme-linked immunosorbent assays (ELISA) were performed using commercially available kits addressing MCP-1, IL-6 and IL-8 (RayBiotech, USA) according to the manufacturer’s instructions. The release of EGF, GM-CSF, IL-1α, IP-10, PAI-1, PDGF-BB, TNF-α and VEGF were determined for HUVEC which were exposed for 24 h to CeO_2_ nanoparticles (100 µg/mL) using Human Mix and Match Customized Cytokine ELISA Strips (Signosis, Inc., USA). The pro-inflammatory impact of SiO_2_ nanoparticles was determined using MCP-1 ELISA kit (RayBiotech, USA). For this purpose, HUVEC were exposed to 30,000 SiO_2_ nanoparticles per cell for 24 h. On the basis of the standard curves, the amounts of released proteins were calculated (fg cytokine/cell).

#### Determination of reactive oxygen species (ROS) after nanoparticle exposure

To assess the oxidative stress after nanoparticle exposure, the activity of reactive oxygen species (ROS) was measured using the OxiSelect™ Intracellular ROS Assay Kit (Green Fluorescence, Cell Biolabs, Inc., USA). Cells were cultured in black 96-well culture plates and treated with CeO_2_ nanoparticles (100 µg/mL) for 24 h or 72 h. Then, the cells were washed with Hank’s BSS and incubated with a 0.1× (100 μM) solution of cell-permeable 2’,7’-dichlorodihydrofluorescin diacetate (DCFH-DA) in cell culture media for 45 min at 37 ºC. In principle, cellular esterases deacetylate the DCFH-DA to non-fluorescent 2’,7’-dichlorodihydrofluorescin (DCFH). ROS oxidize DCFH to fluorescent 2’,7’-dichlorodihydrofluorescein (DCF), which can be detected by fluorescence with a fluorometric plate reader (480 nm excitation, 530 nm emission; TECAN Infinite^®^ M1000 PRO, Tecan Group Ltd., Switzerland). The measured fluorescence intensity is proportional to the ROS levels within the cell cytosol. The obtained ROS levels were normalized to the relative ATP content of the cells to reveal the changes in cell number as result of nanoparticle treatment and incubation time.

#### Determination of relative cellular dehydrogenase activity

The relative cellular dehydrogenase activity of endothelial cells, which were treated with SiO_2_ nanoparticles, was determined after defined incubation times. After washing with Hank’s BSS, cells were incubated with 20 µL/well Cell titer 96 Aqueous One Solution Reagent (Promega GmbH, Germany) in culture medium. The supernatants were used for the absorbance measurement at 492 nm via a microplate reader (Sunrise™, Tecan Group Ltd., Switzerland). Data were normalized to untreated control cell populations and are presented as relative values.

#### Statistical analysis

Data were expressed as means with standard deviation. The analysis of variance model was used to analyze the results (IBM SPSS Statistics, version 20.0, Inc, IBM Company, USA). Differences between different treatment groups were determined via the post hoc Bonferroni test and regarded as statistically significant if P ≤ 0.05.

## Supporting Information

File 1 Additional experimental data.
