# Supplementary material for: Biocompatibility of cerium dioxide and silicon dioxide nanoparticles with endothelial cells
Source: Beilstein J Nanotechnol. 2014 Oct 17;5:1795–807. doi: 10.3762/bjnano.5.190 (PMC4222402; doi:10.3762/bjnano.5.190)
Supplement: File 1 — Additional experimental data. [file Beilstein_J_Nanotechnol-05-1795-s001.pdf]

Supporting Information

for

Biocompatibility of cerium dioxide and silicon dioxide nanoparticles with endothelial cells

Claudia Strobel[1], Martin Förster[2] and Ingrid Hilger*[1]

Address: [1]Department of Experimental Radiology, Institute of Diagnostic and Interventional Radiology I, Jena University Hospital – Friedrich Schiller University Jena, Erlanger Allee 101, 07747 Jena, Germany

[2]Department of Internal Medicine I, Division of Pulmonary Medicine and Allergy/Immunology, Jena University Hospital – Friedrich Schiller University Jena, Erlanger Allee 101, 07747 Jena, Germany

Email: Ingrid Hilger - ingrid.hilger@med.uni-jena.de

* Corresponding author

Additional experimental data

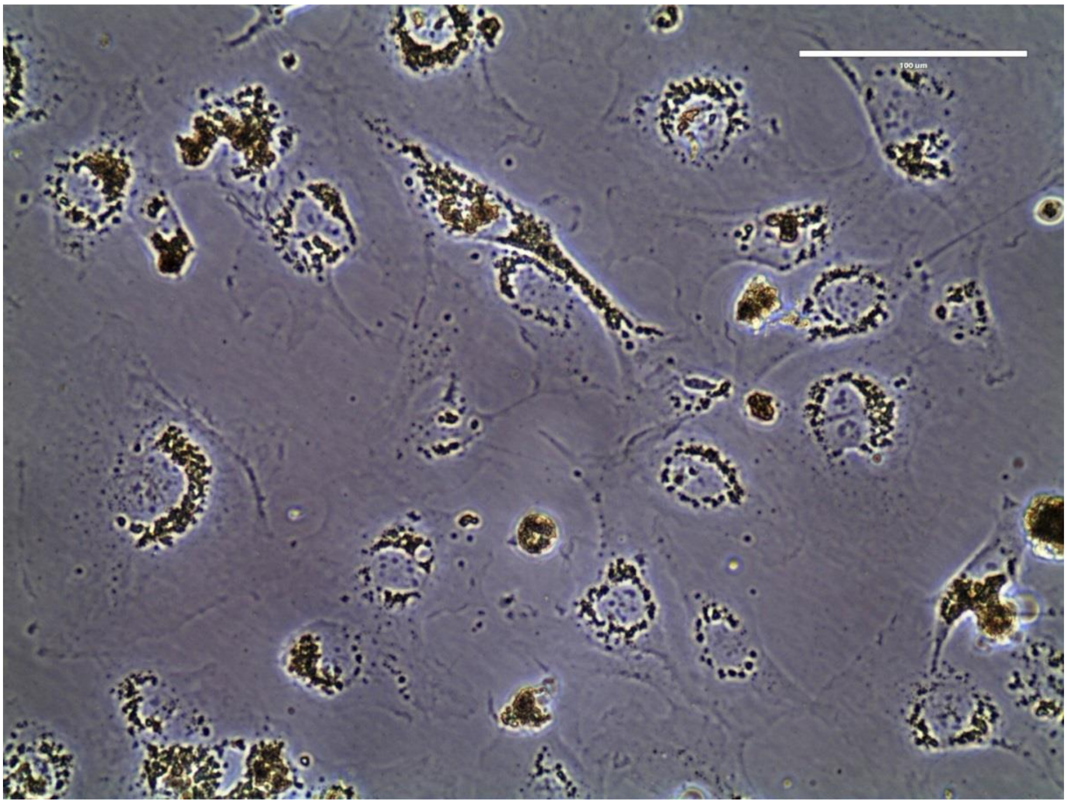

Figure S1: Light microscope image of HMEC-1 treated with 100 µg/mL CeO_2 nanoparticles (sample #B, no fluorescence label) confirmes the peri-nuclear localization.

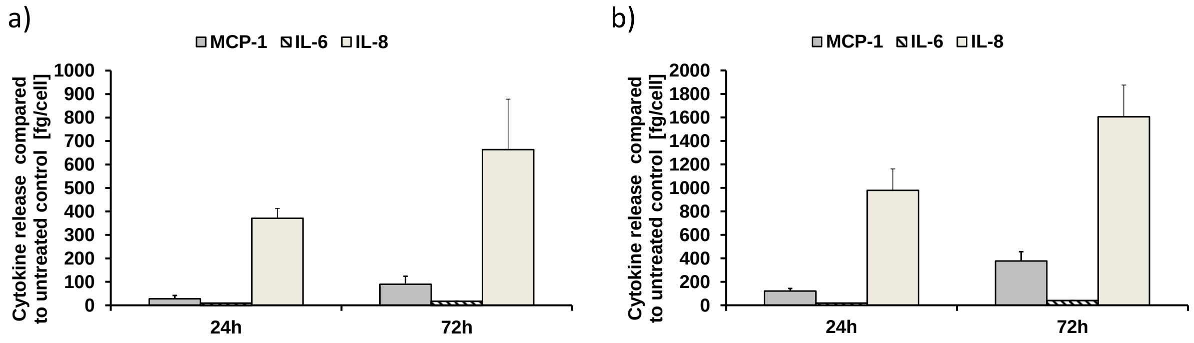

Figure S2: Cytokine release of HMEC-1 (a) and HUVEC (b) after treatment with the positive control IL-1β (c = 2000 pg/ml) at different incubation times (24 h; 72 h; n = 3 independent experiments).

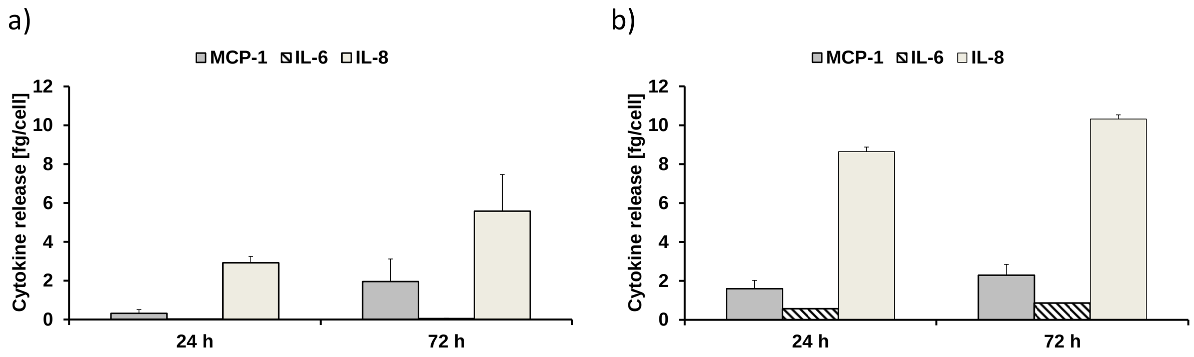

Figure S3: Cytokine release of untreated HMEC-1 (a) and HUVEC (b) at different incubation times (24 h; 72 h; n = 3 independent experiments).
